# Reaching for Precision Healthcare in Finland *via* Use of Genomic Data

**DOI:** 10.3389/fgene.2022.877891

**Published:** 2022-04-26

**Authors:** Tiina Wahlfors, Birgit Simell, Kati Kristiansson, Sirpa Soini, Terhi Kilpi, Marina Erhola, Markus Perola

**Affiliations:** ^1^ Department of Public Health and Welfare, Finnish Institute for Health and Welfare, Helsinki, Finland; ^2^ Department of Information Services, Finnish Institute for Health and Welfare, Helsinki, Finland; ^3^ Management, Finnish Institute for Health and Welfare, Helsinki, Finland; ^4^ Päijät-Häme Joint Authority for Health and Welfare, Lahti, Finland

**Keywords:** genomics, public health, evidence-based design, policy and guidelines, multidiciplinary approaches

## Abstract

Concerns over future healthcare capacity along with continuing demands for sustainability call for novel solutions to improve citizens’ health and wellbeing through effective prevention and improved diagnosis and treatment. Part of the solution to tackle the challenge could be making the most of the exploitation of genomic data in personalized risk assessment, creating new opportunities for data-driven precision prevention and public health. Presently, the utilization of genomic data in the Finnish healthcare system is limited to a few medical specialty areas. To successfully extend the use of genomic information in everyday healthcare, evidence-based and feasible strategies are needed. The national actions that Finland is taking towards this goal are 1) providing scientific evidence for the utility of genomic information for healthcare purposes; 2) evaluating the potential health-economic impact of implementing precision healthcare in Finland; 3) developing a relevant legal framework and infrastructures for the utilization of genomic information; 4) building a national multidisciplinary expert network bringing together relevant professionals and initiatives to achieve consensus among the different stakeholders on specific issues vital for translating genomic data into precision healthcare; 5) building competence and genomic literacy skills among various target groups; and 6) public engagement (informing and educating the public). Taken together, these actions will enable building a roadmap towards the expedient application of genomic data in Finnish healthcare and promoting the health of our citizens.

## Introduction

Similar to many other countries, Finland is getting ready for the era of personalized prevention and care. Overall, Finnish society is undergoing a transition that includes significant structural reforms. The organisation of public healthcare, social welfare, and rescue services is being reformed, resulting in the transfer of the responsibility for organising these services from municipalities to wellbeing services counties from the beginning of 2023 (https://soteuudistus.fi/en/frontpage). The key objective of the reform is to improve the availability and quality of basic public health and social services throughout Finland. Integration of administration, budget lines and health, and social services is the key element of the national reform, which required thorough revision of legislation. Rapid digitalization has brought and continues to bring more tools and resources within the reach of public services, which in the healthcare setting creates novel opportunities for realising the value of scientific breakthroughs and research results.

**FIGURE 1:**
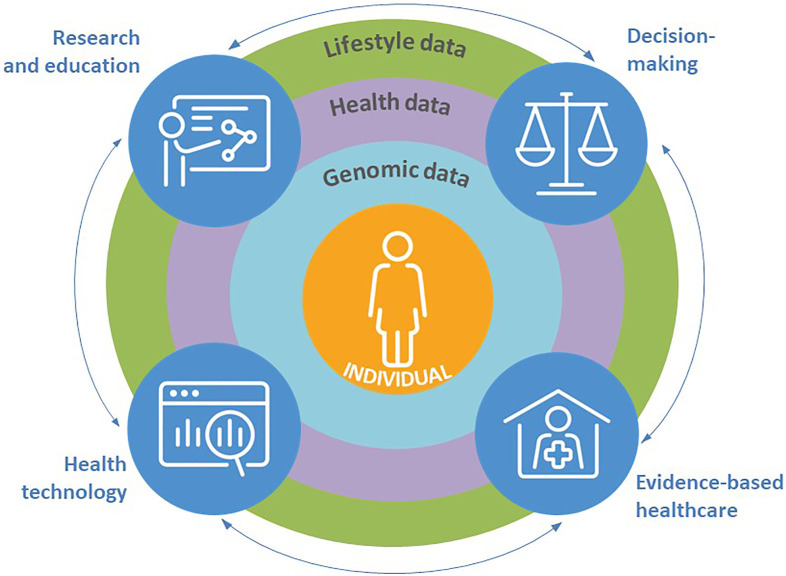
A concept for data-driven precision healthcare in Finland.

There are certain strengths in our country that can be efficiently harnessed for serving precision health care. These include universal health coverage, strong national public health care, reliable national registries, 100% penetrating digital health records, wide-ranging population cohorts and clinical sample collections, and finally the engaged citizens and culture of trust. Advanced technologies offer new digital applications driving the accumulation of individual-level big data (lifestyle, health, and omics data) while simultaneously allowing novel ways for prevention and improved diagnostics and treatment of diseases (Figure 1). The issue of continuous monitoring and evaluation, leading to mid-project adjustments, is something that the Finnish system of science, technology and innovation is well known for and has a central role also in the present endeavour.

The Finnish government desires to achieve transformation of national health care by the utilization of novel technologies and new possibilities offered by scientific research. Our government has launched two health-related national strategies: the National Genome Strategy (2015) and the Health Sector Growth Strategy for Research and Innovation Activities. The related national operations have been combined under the Personalised Medicine Initiative led by the Finnish Ministry of Social Affairs and Health. Among other endeavours, this initiative aims at establishing specific national clusters of excellence, including the national Neurocenter, Cancer Center, Genome Center, and Drug Development Center, as well as harmonizing the national biobank operations.

## National Actions Towards the Concept of Data-Driven Precision Healthcare

**FIGURE 2:**
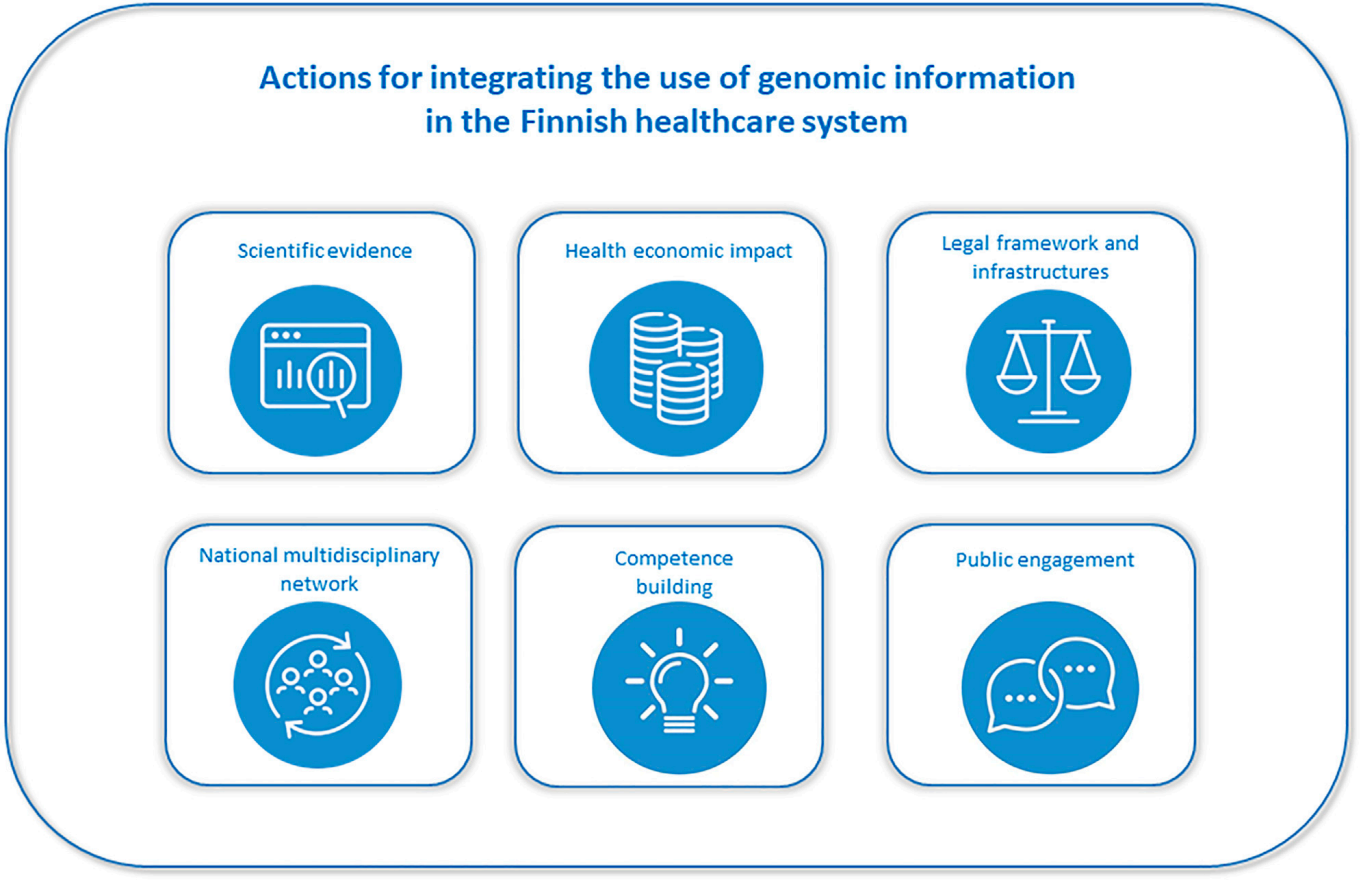
Integrating the use of genomic information in the Finnish healthcare system.

While the concept of data-driven precision healthcare for the utilization of various types of data (e.g., health, lifestyle, omics) is quite universal and parallel, the advancement of functional integration of each data type into the healthcare system will require setting up a relevant working group dedicated to producing a detailed roadmap suitable for the specific data type. A prerequisite for achieving high impact is a carefully planned, nationally coordinated implementation and integration of planned activities with the existing health care structures and functions. In the present article, we focus on the national actions that are being taken to reach a wider and more versatile use of existing genomic data in the Finnish healthcare system (Figure 2).

### Providing Scientific Evidence for the Utility of Genomic Information in Precision Healthcare

Researchers have discovered thousands of genes that harbour variations contributing both to human illnesses and patients’ responses to pharmacotherapies. Some of these discoveries also enable targeting the molecular basis of selected diseases. Despite the huge progress in genomic research over the past decades, there is still need for basic research in the field. It is the responsibility of the medical science community to ensure that any precision healthcare operation planned to be implemented in national health care is ethical, cost-effective, and based on clinically relevant evidence.

To foster its biomedical research, Finland has established a nationwide biobank network, the Finnish Biobank Cooperative—FINBB (https://finbb.fi/en/), which provides researchers with centralized access to the collections and services of Finnish biobanks and their background organizations (Fingenious Digital Service for Researchers, 2022; https://site.fingenious.fi/en/). An excellent example of opportunities created by such an infrastructure is FinnGen (FinnGen research project, 2022; https://www.finngen.fi/en), a large public-private partnership aiming to provide novel medically and therapeutically relevant genomic information that can be utilized in future precision healthcare. FinnGen is a research project that aims to collect and analyse genome and health data from 500,000 Finnish biobank participants. The permissions to utilise the national health register data for research purposes are applied from national authorities. The FinnGen project complies with existing national legislation (in particular the Biobank Law and the Personal Data Act) and will conform to any new laws. The EU Data Protection Regulation that came into force in May 2018 has been taken into account when planning the project.

To obtain credible evidence for the utility of genome data in Finnish healthcare, further comprehensive implementation and intervention studies are needed to fully understand the possibilities and implications of using genomic information as part of clinical care. Several pilot studies on the use of genomic information for assessing a personalized total risk of common chronic diseases have been initialized in Finland and Estonia. The P5-study (Marjonen et al., 2021) recruited 3,177 biobank participants to receive on-line feedback on their total individual risk of coronary heart disease, type 2 diabetes and venous thrombosis. The calculated risk included a polygenic risk score (PRS) and selected clinically important single variants. The GeneRISK-study (Widén et al., 2020) invited 7,342 individuals to receive their PRS-enhanced risk score of cardiovascular disease via a web-based portal. In Estonia, the Estonian biobank has returned genetic results on breast and ovarian cancer (Leitsalu et al., 2021) and familial hypercholesterolemia (Alver et al., 2019). These studies have reported no major issues or mishaps in combing genomic data with more traditional risk factor data, and the information has generally been well received by the study participants. These pilot studies pave the way for larger, preferably randomized studies on personalized prevention of common chronic diseases to investigate whether the clinical and preventive value of genomic information adds healthy life years for the study participants.

The fast increase in the supply and customer-base of private direct-to-consumer commercial genetic testing companies along with certain unsolved issues regarding their business model and data quality (Blell and Hunter 2019; Horton et al., 2019) raises some concerns about future trajectories. If public healthcare does not take a stand on utilizing genomic information in the prevention of common chronic diseases, the field may be left unregulated. This would not only confuse the customers of the commercial genetic testing companies, but also produce unnecessary and in some cases even a harmful burden on the healthcare system, when the customers turn to the health professionals for further advice. Also, it should be noted for the sake of equality, that if some of the commercial genetic tests are of clinical use but not adopted in public healthcare, they remain available only to those with an interest and financial opportunity, which may lead to increased socioeconomic health differences. This underscores the need for gathering further evidence for the utility of genomic information in preventative, personalized healthcare via conducting randomized controlled proof-of-concept studies. The utility of pharmacogenetics has been generally accepted for specific indications, but its wide-spread application to general healthcare is not yet standard practice in our country. However, as a pilot for applying genomics in medicine it is appealing on several fronts. First, most of the genetic variants with pharmacogenetic effects are single polymorphisms, often single nucleotide polymorphisms (SNPs), which makes laboratory detection simpler compared to more complex variants. Secondly, there have been significant scientific advances regarding these variants and there is consensus on the usability of the variant information as well as free on-line access to this information (www.pharmgkb.org). Thirdly, information available on existing pharmacogenomic variations can help in tailoring individual drug dosing and avoid potential adverse drug events.

### Evaluating the Health-Economic Impact of the Wider Use of Genomic Data in Finnish Healthcare

The Finnish national genome strategy emphasizes the importance of analysing the costs and health benefits related to the utilization of genomic data in healthcare. Politicians and policy makers need evidence on how precision public health influences the health of patients and citizens and the costs of healthcare. Good decisions by government require good data from the scientific community. Health economic evaluation through registry- and trial-based statistical modelling generates evidence-based information to facilitate decision-making and appropriate allocation of limited healthcare resources under uncertain conditions. Besides pure economic growth, it is also important to evaluate the societal value of precision healthcare. It is possible that the true economic values of personalised approaches can only be witnessed in the long-term, but there are certain aspects that can be addressed already in the shorter term.

Prior to large-scale introduction of genomics to public healthcare, health economic evaluation, and studies tailored towards specific health systems and populations are required. Pilot projects conducted in a real-life health care setting would provide information on various potential problems, practicalities, and unanswered questions related to the widespread utilization of genomic information. These include the educational needs of the health professionals and managers, adequate information to patients such as the pre- and post-counseling information (written, face-to-face, training/profession of counsellors, information to the relatives), source and logistics for DNA samples (blood, buccal swab, saliva), timing of analyses (e.g., before or after the prescription), and storage of DNA and genetic data and policies to regulate access to them. This all adds to the total costs of introducing genetic information to healthcare at the population level and needs to be considered when evaluating the cost-benefit ratio of any genomic analyses. The articles by Martikainen *et al.* and Marjonen *et al.* in the present issue of this journal touch upon this topic in more detail. There is still need for true clinical pilot studies preferably in randomized settings including continuous health economic evaluation in Finland.

### Developing a Legal Framework and Infrastructures for Using Genomic Data in Finnish Healthcare

While from the social justice perspective the right to health and right to enjoy scientific and technological achievements belong to the key human rights guaranteed by constitutional law, the wide use of genomic data will cause certain challenges related to ethical, legal, and social implications (ELSI). While on the one hand, national regulation should be in place to protect citizens from the misuse of their sensitive data, on the other hand, the same regulation should also enable the use of genomic data for justified purposes. Finland has enacted specific legislation to promote the responsible use of social and welfare data for secondary purposes and biobank activities for the benefit of citizens. Thus, as research and innovation pose significant public interest, legislation needs to balance the rights of both individuals and society. In a welfare society, all citizens should have a universal access to social and health care services based on reliable evidence and technological developments.

In Finland, the preparation of the Genome Act is currently on the homestretch and the new legislation is estimated to be accepted by Parliament in June 2022. The main intention of the Genome Act is to enable responsible, equal, and secure processing of genomic information for the benefit of citizens’ health and well-being. Based on the Genome Act, a national Genome Center will be established in Finland. The envisaged centre will act as a national expert authority outlining future directions and practices related to the exploitation of genomic data. Simultaneously with legislative work, the operation models and information architecture for genomic data has been put together in a separate Expert Working Group drafting a secure flow of genomic data to the healthcare system.

The tasks of the national Genome Center will also include participation in various international networks and collaborations. International sharing of information has been pivotal for the generation of genomic knowledge, and its introduction for clinical use. Thus, submitting genotypes and phenotypes into specific international data bases (e.g., OMIM, ClinVar, ClinGen) and other platforms should be made legally possible for new discoveries. An example of such a platform is Matchmaker Exchange which has been established for detection of rare clinical variants globally. Legislation should support the sharing of variants and related clinical data to accelerate the accumulation and updating of knowledge and interpretation of variants with clinical significance. To achieve optimized patient outcomes via the utilization of novel genomic data, careful outcome-based clinical interventions may be necessary in the future.

### Building a National Multidisciplinary Network to Support the Use of Genomic Data in Healthcare

The successful implementation of precision healthcare in Finland demands political commitment, new thinking, novel approaches, strengthened public-private collaboration as well as strong science policy advice to leadership. Novel collaborations between various stakeholders and coordinated approaches across different levels of society are required. It is crucial to include healthcare leaders and managers in the process to get an adequate commitment to the gradual integration of new activities in the existing public health system. To gain a comprehensive overview of the current situation, the Finnish Institute for Health and Welfare (THL) in collaboration with the Finnish Ministry of Social Affairs and Health has in April 2021 appointed a national Expert Working Group for genomic medicine. The main objective of this working group is to update the genome strategy published originally in 2015. The activity of the working group is organized in three main pillars: 1) Expert Working Group meetings, 2) brainstorming workshops with a larger group of relevant stakeholders, and 3) supporting pilot projects advancing the use of genomic information in the selected specialty areas, for example pharmacogenomics. The activity of the national Expert Working Group is of direct benefit for the upcoming national Genome Center and its future operations.

### Supporting Competence and Genomic Literacy Skills Among Healthcare Professionals

The integration of genomic information and precision medicine in the Finnish healthcare system will require new competencies and skills practically from all representatives across society. Healthcare providers, healthcare managers, policy makers, healthcare professionals, and government officials need to be convinced about the benefits and made aware of the challenges regarding genomics and precision public health. This will demand the involvement of the regional governments as well as investments in education and capacity building.

The article by Halkoaho *et al.* in the present issue of this journal opens the competence and literacy issues in more detail and describes some examples of how the implementation of required competence building in Finland has currently been started.

### Public Engagement

Whilst scientific advances yield innovations that can improve citizens’ lives, there is often a communication gap between the scientists who perform research and the citizens who might benefit from it. Nevertheless, to truly improve the wellbeing of people via the use of genetic information, the general public needs to adopt the proposed novel strategies and therapies. Much effort should be invested in education and awareness training for the public. Also, the initiation of a societal dialogue between the scientific community and the general public will be crucial to successfully empower citizens, demystify sometimes complicated, and confusing terms, technology and other matters of science, as well as limit the potential spread of misinformation. Maintaining the trust and engagement of Finnish citizens is of the utmost importance (Zimani et al., 2021).

## Discussion

Concerns over health and social care capacity in the future along with on-going demands for sustainability have globally generated numerous initiatives and strategies aiming to facilitate the realization of preventive personalized medicine and precision healthcare. One of such initiatives, the International Consortium for Personalized Medicine (ICPerMed), has shared their vision on how personalized medicine will lead to next generation healthcare by 2030 (https://www.icpermed.eu/media/content/Vision_Paper_2019.pdf). ICPerMed refers to a paradigm change that will shift the focus in healthcare from treatment towards risk definition, patient stratification, and personalised health promotion and disease prevention.

By 2030, digital innovation is expected to prompt significant investments in centralized data infrastructures and digital platforms to support data management, interoperability, access, and data sharing between citizens, health professionals and researchers. The increasing use of genomic information will open novel opportunities for tailoring and refining the management of diseases, which is expected to result in a decrease of the more expensive and avoidable diagnostic procedures and treatments. It is crucial to engage people in this transformation and to improve their genomic and health literacy.

Globally, several countries have recently launched national genomic strategies emphasizing the importance of basic and translational research. In the United Kingdom, the nation is strongly committed to a future where genomics will greatly improve the wellbeing of the United Kingdom and worldwide population through the development of a better understanding of the genetic causes of disease, along with the provision of tailored and most suitable therapies to the patients. Also, predictive interventions addressing diseases already before they appear are a central part of the United Kingdom genome strategy. In the United States, the National Human Genome Research Institute (NHGRI) at the National Institutes of Health (NIH) has recently launched a strategic vision for improving human health at the forefront of genomics (https://www.genome.gov/2020SV). In this strategy, they identify future research priorities and opportunities in human genomics, with an emphasis on health applications. The NHGRI strategic vision underlines major unsolved problems that need to be addressed to fulfill the promise of genomics for human health. The NIH also has the *All of Us* research program (All of Research Program, 2022; https://allofus.nih.gov/) working to improve health care through research. The ambitious goal is to build a program of over 1M people living in the United States aiming to discover disease risk factors, tailor therapies more individually, connect people with clinical studies and promote healthy lifestyles. In Australia, The Genomics Health Futures Mission will invest in total $500 million over 10 years in genomic research from 2018–19 to 2027–28. This is expected to improve the testing and diagnosis of many diseases, help to personalize treatment options, improve health outcomes, and reduce unnecessary interventions and health costs. As the mission channels funding to research on integrating genomic knowledge and technology into clinical practice, it will markedly support the implementation of genomics into clinical use.

In Finland, there are still certain challenges to be tackled prior to large-scale utilization of existing genomic information. A major reform of the Finnish healthcare social welfare and rescue services via thorough revision of the guiding legislation has been launched recently and will be in full form in 2023 (https://soteuudistus.fi/en/frontpage). The major change is that the responsibility of these services is transferred from 309 Finnish municipalities to 21 wellbeing service counties. This is the largest single transformation in the Finnish healthcare system since the early 1970’s and the effect of the reform on the ability of the health system to take in novel applications should be strengthened *via* significantly larger health and social care providers. In the future, the local leading civil servants and chief medical officials will have an even stronger role in deciding on the allocation of budget for regional activities. Thus, to promote adequate regional action in the introduction of genomics to public health care, the involvement of these leaders with other regional opinion leaders will be crucial. Finland has a good record for its approach of developing a system and then monitoring it continuously whilst adapting it as it continues over time, the planning and implementation of national health programs into regional activities as an example (Erhola et al., 2019). Such experience now needs to be replicated in introducing genomics to healthcare in a systematic and sustainable way.

There is ample cross-sectional and even prospective evidence for the potential of using genomic data as one of the tools in personalized medicine (Polygenic Risk Score Task Force of the International Common Disease Alliance, 2021; Slunecka et al., 2021). However, as for any novel method to be implemented in clinical practice, it is important to scrutinize the true utility of genomics in precision healthcare. With this said, there is still a need for ample amounts of research. Firstly, we need prospective studies with preventive interventions based on knowledge of genomic and other risk factors where the impact is measured in hard endpoints, such as disease events. Secondly, we need health-economic studies, conducting cost-benefit analyses of the setting, not only including the cost of genetic data, the decision-support systems and training needed for practice, but also the secondary impact of this novel information on the use of health services. Thirdly, we need studies on the ELSI questions and citizens’ perception of the widening use and availability of genomic data. All of these needs call for large studies with tens of thousands of study subjects generating various challenges and high costs that are out of reach at least for any Finnish research funding agency alone. To perform such studies, innovative funding structures such as collaborative public funding, government programs and public-private partnerships will be needed. The data that paves the way for taking a next step toward genomic medicine already exist. This next step would require a robust strategy that will lead to policies and programmes providing the appropriate tools for implementation.

## Data Availability

The original contributions presented in the study are included in the article/Supplementary Material, further inquiries can be directed to the corresponding author.
